# Electronic healthcare applications and programs among healthcare workers in Riyadh and conflict management

**DOI:** 10.1016/j.jtumed.2021.11.016

**Published:** 2022-01-22

**Authors:** Wafa A. Alhazri, Bussma A. Bugis

**Affiliations:** a Department of Public Health, College of Health Sciences, Saudi Electronic University, KSA; b Department of Dentistry, Northern Al Nadeem Primary Healthcare Center, Second Health Cluster, Riyadh, KSA

**Keywords:** إدارة الصراع والرعاية الصحية, تطبيقات الكترونية, برامج الكترونية, المملكة العربية السعودية, Conflict management, Electronic applications, Electronic programmes, Healthcare, KSA

## Abstract

**Objective:**

Electronic healthcare applications and programmes enable the use of computers, networks, and information technology to improve healthcare quality and patient safety, and secure confidential access to health information in order to enable individuals and communities to make the best possible health decisions. We study conflicts challenging users of e-health electronic applications and programmes in Riyadh.

**Methods:**

We use a cross-sectional descriptive design to target all healthcare professionals who interact with e-health applications and programmes. Healthcare providers took a questionnaire survey online.

**Results:**

Of the 169 responses to our questionnaire, 78.1% are female, and 46.2% are aged between 31 and 40 years. As many as 59.2% always use these applications, and 33.7% use them occasionally. Only 7.10% of the participants rarely or never use them. As many as 31.4% recognized that these applications led to conflicts at the workplace. Of these, 50.6%, 13.6%, and 35.8% stated that they caused decision-related, ethical, and other types of conflicts, respectively.

**Conclusion:**

We conducted this study among healthcare providers in Riyadh, KSA, and found that the use of e-health applications and programmes encountered some difficulties.

## الملخص

**أهداف البحث:**

تتيح التطبيقات والبرامج الصحية الإلكترونية استخدام أجهزة الكمبيوتر والشبكات وتكنولوجيا المعلومات لتحسين جودة الرعاية الصحية وسلامة المرضى وتأمين الوصول السري إلى المعلومات الصحية وذلك يساعد الأفراد والمجتمعات من اتخاذ أفضل القرارات الصحية الممكنة. لذلك قمنا بدراسة قضايا الصراع التي تواجه مستخدمي التطبيقات والبرامج الإلكترونية للرعاية الصحية في الرياض.

**طرق البحث:**

استخدمنا في هذه الدراسة تصميما وصفيا مقطعيا لاستهداف جميع المتخصصين في الرعاية الصحية الذين يتعاملون مع التطبيقات والبرامج الإلكترونية للرعاية الصحية. قمنا بتوزيع استبيان معد لمقدمي الرعاية الصحية عبر الإنترنت.

**النتائج:**

جمعنا 169 عينة من المشاركين في الاستبيان. مثلت الإناث 78.1٪، و46.2٪ من المشاركين تتراوح أعمارهم بين 31-40 سنة. وجدنا أن 59.2٪ من المشاركين أشاروا إلى أنهم استخدموا هذه التطبيقات دائما، و33.7٪ من المشاركين استخدموها أحيانا، و7.1٪ فقط من المشاركين أشاروا إلى أنهم نادرا ما يستخدمونها أو لم يستخدموها مطلقا. اعترف 31.4٪ من المشاركين بأن هذه التطبيقات أدت إلى بعض النزاعات في العمل، وقال 50.6٪ منهم أنها تسببت في نزاع في القرار، و13.6٪ كشفوا عن صراعات أخلاقية، و35.8٪ أشاروا إلى أنواع أخرى من النزاعات.

**الاستنتاجات:**

أجريت الدراسة الحالية في مقدمي الرعاية الصحية في منطقة الرياض بالمملكة العربية السعودية ووجدنا أن استخدام التطبيقات والبرامج الإلكترونية في الرعاية الصحية واجهت بعض الصعوبات. وبالتالي يجب الاستمرار في تطوير الوضع الحالي.

## Introduction

The interest in e-health applications and programmes is increasing in KSA. They enable the use of computers, networks, and information technology (IT) to improve the quality of healthcare services and patient safety, and secure confidential access to health information in order to enable individuals and communities to make the best possible health decisions.^1^ E-healthcare applications enhance the sharing of patient information and increase contact among professionals.^2^ The introduction of health information technology (HIT) within a complex adaptive healthcare system plays a significant role in improving the care provided to patients. However, it has unintended consequences and introduces new challenges.^3^

For several years, the scientific community has sought to understand the complex interactions among people, the environment, and technologies in order to safely develop, implement, and maintain new digital applications that are safe for patients. However, these applications may produce new challenges to patient safety.^4^ Therefore, to increase the efficacy and safety of e-health applications, a shared responsibility and sociotechnical approach are essential in order to focus on people, processes, the environment, and technology.^5^ E-health applications must be technologically advanced to support user goals and workflows, and organisations must apply them.^3^ Given the vast amounts of health data these applications collect, they have the potential to help transform healthcare and increase its quality and efficiency.

KSA unveiled Vision 2030 on April 25, 2016, along with a plan of action to transform its economy through the development and creation of alternative sources of income. The National Transformation Program 2020 (NTP) was announced in June 2016 as an interim measure to lay the foundation for achieving the aims of Vision 2030.^6^ Healthcare was one of the areas of growth identified in Vision 2030, with the aim of developing the private sector and reducing reliance on the public healthcare system. The NTP established a set of national priorities and goals to be achieved through job creation, the strengthening of partnerships in the private sector, maximisation of domestic industries, and digital transformation. One of the priorities and goals under the NTP is to be achieved by improving the efficiency and effectiveness of the healthcare sector through the use of IT and digital transformation.

The healthcare delivery system in KSA is changing at a rapid pace to keep up with the healthcare needs of the growing population and to improve the management of chronic diseases. However, the use of e-health applications and programmes are fully achieved and experienced by healthcare professionals who are satisfied with their results.^7^ Some unintended consequences with patient safety implications from the same systems result in moral distress and conflicts among healthcare professionals.^8^ These conflicts include ethical issues, which require ethical decision-makers to increase the efficacy and safety of these applications. Conflict management is critical for organisational effectiveness and efficiency and healthcare professionals must manage conflict in order to provide an environment that improves personal growth and ensures the quality of patient care.^9^ Despite the importance of these topics, very little is known about the situation in KSA, especially Riyadh. Therefore, this study investigated the conflicts that challenge the users of e-health applications and programmes in Riyadh. It assessed the levels of satisfaction with e-health applications and programmes among healthcare workers, and the extent to which they adapt to healthcare electronics. It also determined the most appropriate solutions for these conflicts.

## Materials and Methods

This cross-sectional descriptive study examined healthcare professionals who interacted with e-health applications and programmes in Riyadh, KSA. Healthcare professionals who dealt with e-health applications and programmes, and worked for any healthcare facility in Riyadh were eligible to participate. An online questionnaire was developed based on a literature review and distributed to healthcare professionals in Riyadh. The questionnaire comprised three sections. The first section gathered data on the demographic factors of participants including their age, gender, duration of experience, department, and profession. The second section assessed the satisfaction of participants with the use of e-health applications and programmes. It comprised 15 positive questions on the use of an e-health application. Responses were sought on a five-point Likert scale where 1 = strongly agree and 5 = strongly disagree. The questionnaire was distributed through email invitations and different social media channels. The survey was conducted between March 28 and April 27, 2021. Participants were given the option to participate in Arabic or English.

A simple random convenience sample comprising 169 participants was collected. Each observation was converted to numerical values for analysis, where 1 = strongly disagree and 5 = strongly agree. Total scores were calculated and ranged from 15 to 100. Higher scores meant higher levels of satisfaction. The last secton comprised 12 questions, of which 10 assessed the level of conflict, and 2 sought to identify solutions to conflicts.

Microsoft Excel 2010 was used for data entry. Data analyses were performed using Statistical Package for Social Sciences (SPSS) version 22.^10^ Descriptive statistics were performed for all study variables, which included the measures of prevalence, means, standard deviation, and shape of distribution. Chi-squared and t-tests were used to assess the associations between variables. All statements were considered significant when the p-value was confidence level of 95%.

All participants were informed of the objectives of the study. Participation was voluntary and informed consent was embedded at the beginning of the online questionnaire. Anonymity was guaranteed by assigning each response a code number for analysis.

## Results

**Figure 1:**
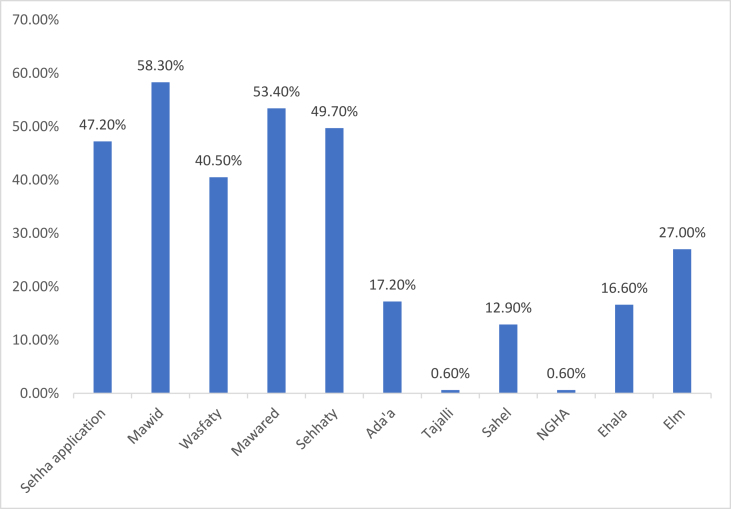
Frequency of using of different electronic health applications.

A total of 169 survey responses were collected (response rate = 60.7%). Among the participants, 78.1% (132 participants) were women, and 21.9% (37 participants) were men. Further, 46.2% were aged between 31 and 40 years, 26.6% between 41 and 50 years, 16.6% between 20 and 30 years, and 10.7% between 51 and 60 years. A total of 55.6% indicated that they had more than 10 years of experience, 38.5% had between 3 and 10 years of experience, and 5.9% had less than 3 years of experience (Table 1). As many as 37.9% worked for the MOH, 26% worked in the primary medical healthcare sector, 21.3% worked in military healthcare facilities, and the rest worked in hospitals in health clusters. Further, 65.1% reported that they were in clinical professions, working as physicians, nurses, or pharmacists, 23.1% were in administrative positions, and 9.50% were in technical professions (Table 1). As many as 59.2% indicated that they had always used e-health applications, 33.7% used them sometimes, and only 7.10% rarely or never used them. As many as 56.2% indicated that the phone was the easiest device to use while accessing electronic applications and programmes in healthcare over computers (43.8% participants) (Table 1). Mawid was the most common e-health application and was used by 58.3% of the participants, followed by the Mawared, which was used by 53.4% of the participants. Sehhaty was used by 49.7% of the participants, Sehha by 47.2%, and Wasfaty by 40.5%. Other applications included Elm (27.0%), Ada'a (17.2%), Ehala (16.6%), and Sahel (12.9%) (Figure 1).

**Table 1:** Participants’ characteristics.

Items		n	%
Age	20–30 years	28	16.6
	31–40 years	78	46.2
	41–50 years	45	26.6
	51–60 years	18	10.7
Year of experience	<3 years	10	5.90
	3–10 years	65	38.5
	>10 years	94	55.6
Work place	Ministry of Health	64	37.9
	Primary Medical Centres	44	26.0
	Military Healthcare Facilities	36	21.3
	Riyadh Health Clusters	25	14.8
Type of work	Clinical	110	65.1
	Technical	16	9.50
	Administrative	39	23.1
	Clinical and administrative	3	1.80
	Other	1	0.60
Frequency of using e-health applications and programmes	Never	1	0.60
	Rare	11	6.50
	Sometimes	57	33.7
	Always	100	59.2
Easiest device for using e-health applications and programmes in healthcare	Phone	95	56.2
	Computer	74	43.8

**Figure 2:**
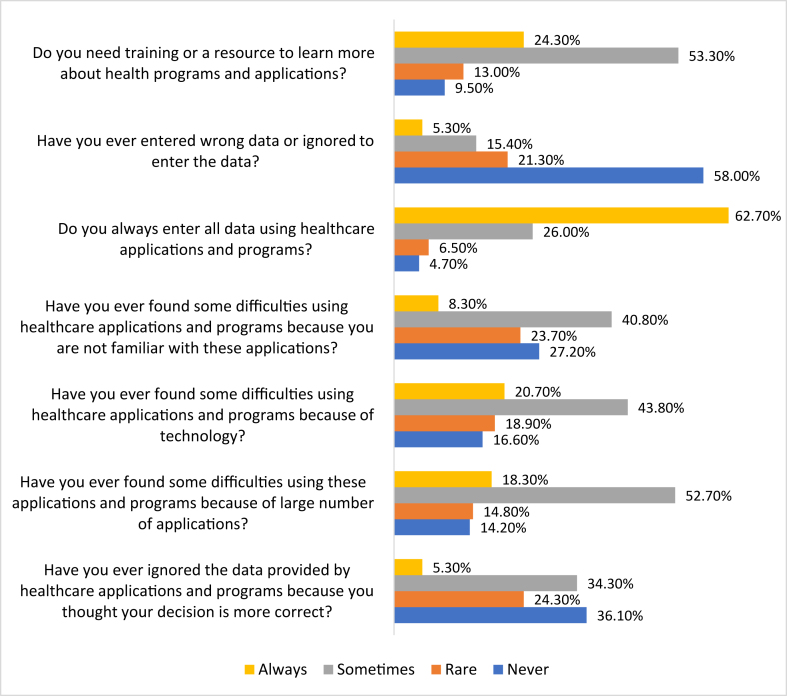
Participants’ difficulties of using healthcare applications and programs.

Table 2 shows the participants' degree of satisfaction with the use of electronic applications and programmes. Most participants agreed that these application and programme systems enhanced their commitment to work (48.5% agreed and 25.4% strongly agreed). A total of 46.7% and 23.1% of participants agreed and strongly agreed, respectively, in signifying their satisfaction with the use of these healthcare applications in making clinical decisions, and 9.50% disagreed with this statement. Considering the ease of using these applications, it was surprising that 26.0% and 27.2% of participants strongly disagreed and disagreed, respectively, on the ease of using these applications, and only 13.0% reported that the use of these applications was easy. As many as 66.8% of the participants were satisfied with the e-health application and programme performance overall, whereas 8.9% were not satisfied. Further, 69.2% were satisfied with e-health application and programme information, and only 57.4% were satisfied with the technical support for these applications. Only 31.4% of the participants found that these applications led to some conflict in work, whereas 50.6% reported that they caused decision conflict, 13.6% reported ethical conflicts, and 35.8% reported other types of conflict. A total of 29.6% of the participants experienced a conflict between their own decisions and what the healthcare applications and programmes advised, whereas 53.8% did not experience this conflict (Table 3). Further, 62.7% reported that they always entered all data using healthcare applications, and 34.3% indicated that they sometimes ignored the data provided by the programmes because they thought that their decision was more appropriate. Almost two-thirds of the participants encountered difficulties in using E-health applications because of the use of many different applications, 64.5% had difficulties because of reasons related to technology, and 49.1% had difficulties because of the lack of familiarity with these applications. As many as 53.3% and 24.3% of the participants thought that they sometimes and always, respectively, needed training or resources to learn more about e-health programmes and applications (Figure 2).

**Table 2:** Distribution of participants based on their satisfaction with e-health applications (%).

Item	Strongly disagree	Disagree	Neutral	Agree	Strongly agree
The e-health applications and programmes system has enhanced my commitment to work	0.60	7.70	17.8	48.5	25.4
I am satisfied with the e-health applications and programmes I use to make clinical decisions	0.60	9.50	20.1	46.7	23.1
I am generally satisfied with the e-health applications and programmes I use	0.60	10.1	12.4	50.3	26.6
With the e-health applications and programmes, my performance at work is satisfactory	1.20	7.10	18.3	50.3	23.1
With the e-health applications and programmes I use, I am very satisfied with my clinical work	1.80	4.10	24.9	46.7	22.5
The e-health applications and programmes motivate me to work hard	2.40	3.60	18.3	50.3	25.4
The e-health applications and programmes system enables effective communication of clinical information to patients	0.60	4.70	19.5	49.1	26.0
The e-health applications and programmes enable the effective capture and storage of patient data	0.60	4.10	14.2	46.7	34.3
The e-health applications and programmes facilitate clinical information-sharing among different parties	1.80	7.70	10.1	47.9	32.5
The e-health applications and programmes increase the quality of documentation	2.40	3.60	9.50	46.2	38.5
It is easier and faster to use e-health applications and programmes than paper	26.0	27.2	10.1	23.7	13.0
Benefits to quality of care are more than expected	0.60	3.00	20.1	52.1	24.3
Overall satisfaction with e-health applications and programme performance	3.60	5.30	24.3	47.3	19.5
Overall satisfaction with e-health applications and programme information	0.60	7.10	23.1	48.5	20.7
Overall satisfaction with technical support	4.10	12.4	26.0	39.1	18.3

**Table 3:** Experiencing conflict in the workplace as a result of using e-health applications.

Item		n	%
Do e-health applications and programmes cause new conflicts at work?	No	72	42.6
	Yes	53	31.4
	I do not know	44	26.0
If yes, what type of conflict?	Ethical conflict	11	13.6
	Decision conflict	41	50.6
	Other	29	35.8
Have you experienced a conflict between your own decision and what the e-health applications and programmes told you?	No	91	53.8
	Yes	50	29.6
	I do not know	28	16.6

Table 4 shows that gender did not significantly affect the level of satisfaction of the participants with the use of e-health applications. However, men seemed to have higher levels of satisfaction. Age had a significant impact, where participants aged between 41 and 50 years had the highest level of satisfaction, followed by those aged between 20 and 30 years. Those who worked at military healthcare facilities had the lowest levels of satisfaction with e-health applications. Technical workers had the highest level of satisfaction, but participants who reported having clinical and administrative positions together showed the lowest levels of satisfaction. Participants who were not satisfied with these applications often refused to undergo any training on using them.

**Table 4:** The association between demographic factors and level of satisfaction (Mean values).

Items		Satisfaction with e-health applications and programme performance	Satisfaction with e-health applications and programme information	Satisfaction with technical support	Overall satisfaction
Gender	Male	2.89	3.00	2.65	44.00
	Female	2.70	2.77	2.52	41.80
	***P-Value***	0.273	0.144	0.524	0.260
Age	20–30	2.89	2.93	2.93	44.4
	31–40	2.51	2.64	2.19	40.0
	41–50	3.02	3.04	2.84	45.56
	51–60	2.78	2.83	2.78	40.56
	***P-Value***	0.026∗	0.076	0.001∗	0.021∗
Years of experience	<3 years	3.30	3.30	3.30	50.1
	3–10 years	2.40	2.57	2.22	38.7
	>10 year	2.91	2.94	2.70	43.94
	***P-Value***	<0.001∗	0.005∗	0.001∗	<0.001∗
Work place	Ministry of Health	2.87	2.83	2.66	43.6
	Primary Medical Centres	2.73	2.84	2.61	43.4
	Military Healthcare Facilities	2.36	2.69	2.14	37.7
	Riyadh Health Clusters	2.96	2.92	2.76	43.3
	P-Value	0.038∗	0.774	0.063	0.041∗
Type of work	Clinical	2.66	2.77	2.44	42.08
	Technical	3.25	3.19	3.06	46.69
	Administrative	2.82	2.87	2.72	43.03
	Clinical & administrative	1.33	1.33	1.33	13.33
	***P-Value***	0.007∗	0.006∗	0.017∗	<0.001∗
Need for training to use e-health programmes and applications	Never	1.88	2.56	1.75	37.1
	Rare	2.77	3.05	2.55	42.5
	Sometimes	2.79	2.77	2.58	42.1
	Always	2.95	2.90	2.80	44.7
	***P-Value***	0.001∗	0.305	0.008∗	0.108

∗Statistically significant at a = 0.05.

## Discussion

Technological advancements have made it possible to develop e-health solutions to make the sharing of health resources more efficient and flexible when compared to traditional healthcare systems, where the exchange of health information is mostly paper-based.^11^ Since its inception, the massive use of the Internet, especially in developed countries, has given rise to new technologies in almost every sphere of life,^12^ of which one is healthcare. Internet technologies have made great strides in telemedicine and telehealth, and these technologies are present in all modern healthcare institutions.^13^ E-health has become a paradigm in the field of telemedicine that encompasses the concepts of health, technology, and trade; and commerce and technology are tools in the service of health.^14^ Chang Liu defined e-health applications as software applications that provide tools, processes, and communications to support e-health practice.^15^ With the advent of wireless communication, there are no longer any spatial or temporal barriers between healthcare providers and patients.^16^ New wireless communication technologies such as mobile communications networks (2.5G, 3G, 4G, and HSPA +), wireless local area networks (WLANs), and wireless personal area networks (WPANs), including Bluetooth, ZigBee, and wireless cellular area networks (WBANs), have strengthened wireless sensing (WSNs), radio frequency identification (RFID), and global reach of microwave access (WiMAX) which are largely used in telemedicine and e-health.^17^, ^18^, ^19^, ^20^, ^21^

We assessed the prevalence of the use of medical applications, the satisfaction of healthcare providers with these applications, and major conflicts related to their use. Most participants preferred smartphones over computers while accessing medical information, 59.2% of them used e-health applications. Aljohani studied healthcare providers in KSA and found that 65% had used smartphones at work, and 30% had used them for work purposes.^22^ Other studies also showed an increase in the use of smartphone applications among healthcare professionals as a training and information tool,^23^^,^^24^ but some studies have found that the prevalence of smartphone use ranged between 66 and 90% in 2012.^25^ The number was higher in studies conducted in 2020 because of the COVID-19 pandemic.^26^^,^^27^ Abolfotouh found that 6.1% of all healthcare providers always used these applications in their practice, and 26.2% of the providers used them sometimes.^26^

Most participants in our study showed a high degree of satisfaction with the use of smartphone applications, except for two main factors, difficulty in use and technical support. Jamal found that 52% of participants reported facing some technical difficulties while using smartphones primarily because of the short battery life. However, most participants reported that smartphones were useful for staff communication and in the consultation and review of patients’ laboratory/radiology results.^27^ Many clinicians consider medical or e-health applications important in finding information, learning, and providing advice quickly. Man found that a mobile application was effective in increasing confidence in treating depression and educating clinicians.^28^ However, future research is necessary to assess the effectiveness and impact of mobile applications in medical and postgraduate education.^28^ Marinkovic stated that portable devices provide opportunities to increase the proficiency of medical students and residents.^19^ Jane and Kim described a three-stage evaluation of a ‘smartphone health app’ device. They concluded that the evaluation tool developed and tested in their study was appropriate and widely applicable in evaluating mHealth applications to determine whether they were reliable and useful.^29^

However, despite the importance of using medical applications and programmes as seen in the literature review, some conflicts emerged as a result of such use. Hawkes found that 43.8% of physicians thought that the use of smartphones during a medical examination would distract from providing appropriate patient care, and 42.5% thought that the use of such applications would harm patients.^23^ We reviewed three types of conflict that may occur while using e-health mobile applications during work: decision-related and ethical conflicts, and conflicts because of difficulty experienced in using them. Only 31.4% of participants found that these applications led to some conflict at work, whereas 50.6% reported that they caused decision conflict, 13.6% ethical conflicts, and 35.8% other types of conflict. Decision conflict was the most common. Decision conflict may occur because the used applications may recommend some decisions that may not in compact with the decision of healthcare providers which are based on medical sciences and practice. Another reason is that the decisions reported in the applications by one healthcare provider may contrast with those reported by another.^30^ In such cases, we found that approximately one-third of the participants left the decision to managers. However, when the conflict occurred as the decision of the e-health programme was different from that of the healthcare provider, 34.3% of the participants indicated that they ignored the data provided by the programmes because they thought that their decisions were correct. This indicated that most healthcare providers did not trust these applications completely. Nearly two-thirds of the participants encountered difficulties because of the large number of applications available. Whereas 64.5% reported difficulty because of the technology, 49.1% encountered difficulty because they were not familiar with the applications. As many as 53.3% and 24.3% thought that they sometimes and always, respectively, needed training or resources to learn more about e-health programmes and applications. Thus, there is a need for training programmes to provide healthcare providers with information on these applications so that they can use them quickly and receive advice on the applications they need to use. In a study that aimed to understand potential factors contributing to the use of Sehha (an application) and determine whether there were technical issues affecting access, satisfaction, and efficiency, the authors found that the users of the mobile application had a better experience with e-health services than the users of the traditional providers in terms of ease of access, satisfaction, and efficacy (measured by number of required doctor visits). The study also showed that 26% of the Seha users reported having technical problems while using the application for the first time, and only 17% reported facing technical problems continuously.^30^

Ethical conflicts were reported by 13.6% of the participants. Ethical problems that are related to the use of mHealth applications including: first, threats to health equity due to inappropriate development of applications; second, threats to privacy of patients, third, reduction of doctor-patients’ relationships; and finally, the problem of chosen mHealth over traditional care because of economic purposes.^31^

We found that conflict because of difficulties in using mHealth applications affect many participants for different reasons including large number of different applications, difficulties with technology, and not being familiar with e-health applications. Some participants needed training or resources to learn more about the health programmes and applications.

Other conflicts include system and vision conflicts. According to Liedner et al.,^32^ system conflicts occur when values understood in a specific IT context oppose those held by group members that use or are supposed to use this system. The theory of IT culture conflict was conducted to examine the contradiction of values in the form of a conflict; where the values when embedded in a system supported the using groups' values, then the culture would remain undetectable.^32^

Our study found that age had a significant effect, with participants between the ages of 41 and 50 years being the most satisfied, followed by those between the ages of 20 and 30 years. Studies have showed that young people used mobile applications better.^33^ However, one study found that elderly patients had high motivation to use electronic counselling services.^34^ Our study found that gender did not significantly affect the participants’ satisfaction with e-health applications. However, men seemed more satisfied overall. Haluza found that gender was also significantly affected by online activities and the adoption of healthy technologies,^35^ which is consistent with the results of another study.^36^

Our study is the first to assess the use of e-health programmes and applications among healthcare providers and the conflicts that occurred because of the use of these applications. However, it also has a few limitations. The first is the dependence on a self-reported questionnaire to collect data as it increases the risk for personal bias. Some participants reflected their experiences while completing the questionnaire. The convenient random distribution of the questionnaire may have led to some response bias. In addition, our study did not cover the technical utilisation techniques of health information exchange that may affect the use of electronic applications and programmes. The use of advanced artificial intelligence and machine-learning techniques was not examined. Future research must address the utilisation of specific technical aspects of e-health programmes and applications.

## Conclusions

A questionnaire-based study was conducted to assess the conflicts involved in the use of e-health programmes and applications among healthcare workers in Riyadh, KSA. The main results of this study are as follows. First, most participants indicated the use of e-health applications and programmes. Second, most participants indicated that they were satisfied with the use of these applications and programmes. Third, approximately one-third of healthcare providers thought that e-health applications and programmes may cause some conflict during work, and identified the main conflict as decision conflict. Finally, difficulties in using these applications included technical problems owing to the large number of applications and lack of familiarity with these application or programme.

There is a need to prepare and conduct training programmes to increase the awareness of e-health applications and programmes among healthcare providers. Overarching programmes that combine the functions of several programmes are necessary. Internet and technical support for devices in health organisations and their maintenance should also be provided. Accountability and penalties for negligence and wrong data entry should be imposed. Finally, as two-thirds of the participants were overwhelmed with the number of applications available, developing hospital policies and guidance on the applications to use and not use are recommended.

## Source of funding

This study did not receive any grant from any funding agency in the public, commercial, and not-for-profit sectors.

## Conflict of interest

The authors have no conflict of interest to declare.

## Ethical approval

The Central Institutional Review Board at the Saudi Ministry of Health (Central IRB-MoH) reviewed and approved the study protocol as log # 2021-38M on 28th March 2021.

## Authors contributions

WAA conceived and designed the study, conducted research, collected, organised, analysed, and interpreted the data, and wrote the initial draft of the article. BAB critically reviewed and revised the content, provided logistic support, and wrote the final draft. All authors have critically reviewed and approved the final draft and are responsible for the content and similarity index of the manuscript.
